# Role of Papain-Like Cysteine Proteases in Plant Development

**DOI:** 10.3389/fpls.2018.01717

**Published:** 2018-12-04

**Authors:** Huijuan Liu, Menghui Hu, Qi Wang, Lin Cheng, Zaibao Zhang

**Affiliations:** ^1^Henan Key Laboratory of Tea Plant Biology, Xinyang Normal University, Xinyang, China; ^2^College of Life Science, Xinyang Normal University, Xinyang, China

**Keywords:** papain-like cysteine proteases, programmed cell death, immunity, stress responses, senescence

## Abstract

Papain-like cysteine proteases (PLCP) are prominent peptidases found in most living organisms. In plants, PLCPs was divided into nine subgroups based on functional and structural characterization. They are key enzymes in protein proteolysis and involved in numerous physiological processes. In this paper, we reviewed the updated achievements of physiological roles of plant PLCPs in germination, development, senescence, immunity, and stress responses.

## Introduction

Proteases include diverse families (e.g., cysteine-, serine-, aspartic-, metallo-, and threonine-proteases) and play crucial roles in protein proteolysis (van der Hoorn, 2008). Based on the evolutionary relationships, they have been subdivided into 61 clans of 253 families (Rawlings et al., 2016). Among them, papain-like cysteine proteases (PLCPs), featuring a nucleophilic cysteine thiol at the active site (i.e., Cys, His, and Asn), are one of the most abundant groups of cysteine proteases (Rawlings et al., 2010).

**FIGURE 1:**
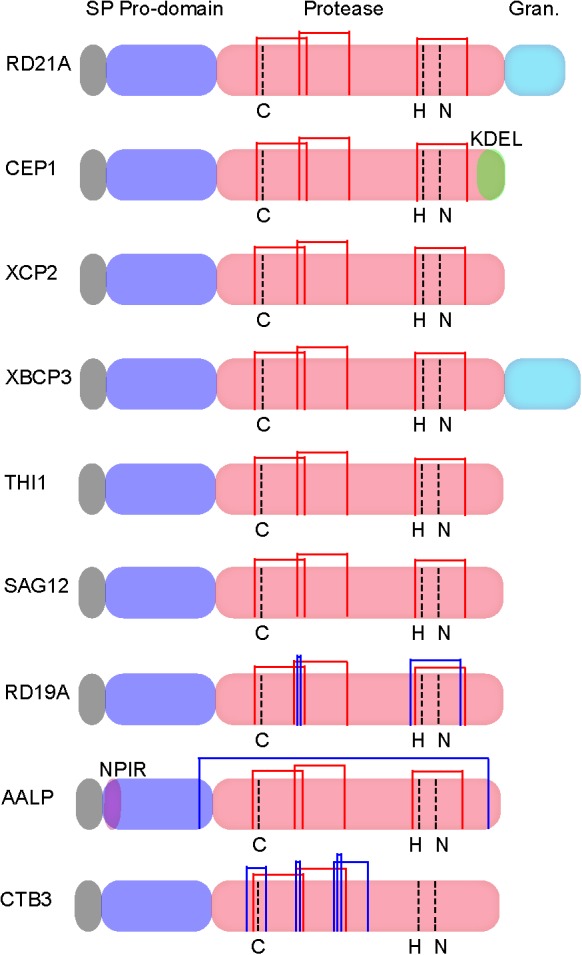
Protein structures of Arabidopsis papain-like cysteine proteases (PLCPs) [modified from Richau et al. (2012) and Misas-Villamil et al. (2016)]. SP, N-terminal signal peptide; Pro-domain, an auto-inhibitory domain; Protease, the catalytic domain contains the catalytic triad Cys-His-Asn; Granulin, C-terminal granulin domain; NPIR, a vacuolar targeting signal; KDEL, a C-terminal retrieval signal for ER localization. Common disulphide bridges and subfamily specific disulphide bridges are indicated with red thin lines and blue thin lines, respectively.

Papain-like cysteine proteases are found in most organisms, including virus (Rawlings et al., 1992), bacteria (Kantyka et al., 2011), yeast (Enenkel and Wolf, 1993), protozoa, plants, and animals (Rawlings et al., 2010; Novinec and Lenarcic, 2013). These enzymes are produced as inactive precursors with a signal peptide for protein secretion and an auto-inhibitory prodomain to prevent unwanted protein degradation (Figure 1; Coulombe et al., 1996). The active protease domain contains the catalytic triad Cys-His-Asn (Figure 1).

**FIGURE 2:**
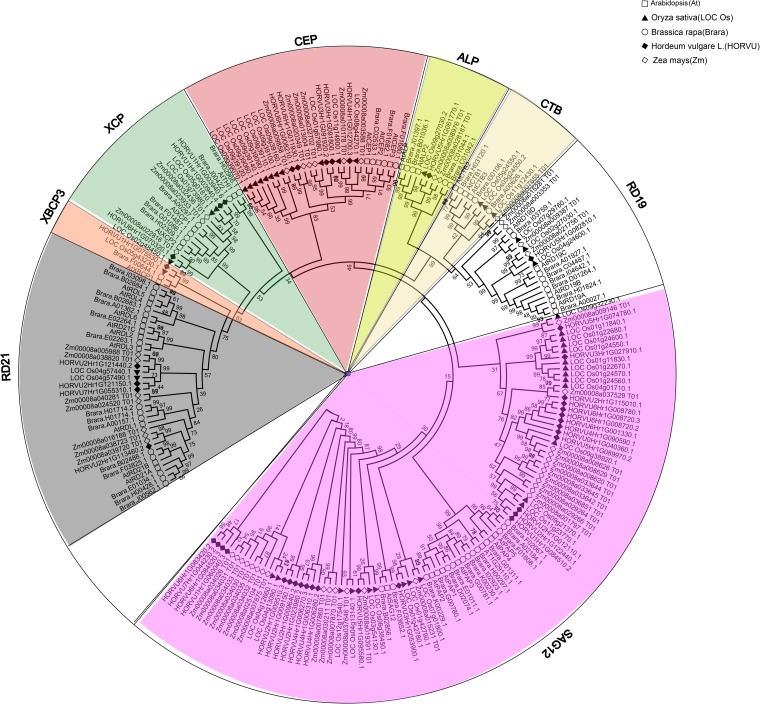
Phylogenetic analysis of PLCPs in Arabidopsis, rice, maize, barley, and *Brassica rapa*. Sequence alignment was performed using MUSCLE and the phylogenetic tree was constructed using bootstrap maximum likelihood tree (1000 replicates) method of MEGA6.

**FIGURE 3:**
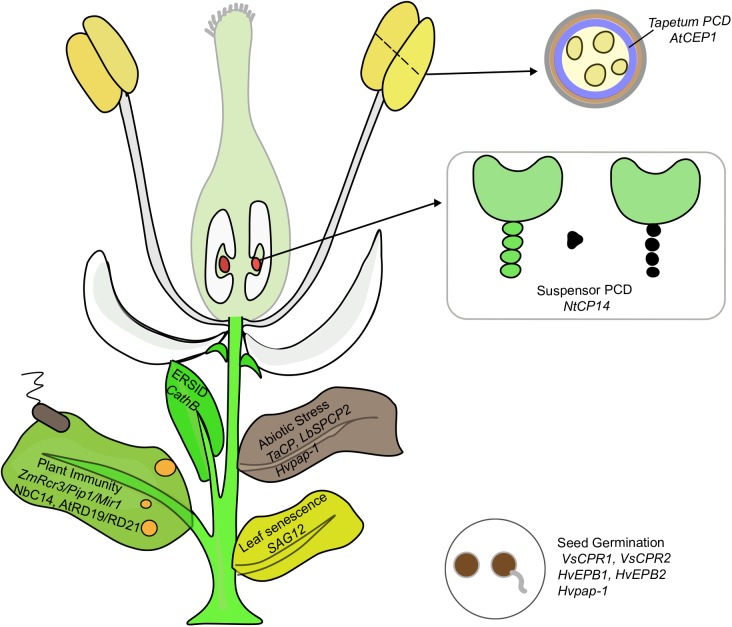
PLCPs play important functions in multiple processes of plant growth including seed germination, PCD, abiotic stress and immunity. The genes implicated in each process are discussed in this review.

Papain-like cysteine proteases genes belong to a large multigenic family with 31, 43, 40, 26, 40, and 24 PLCP family members were identified in Arabidopsis, rubber, cassava, castor, poplar, and grapevine, respectively, and they were divided into 9 subfamilies based on structural characteristics (Figures 1, 2; Martinez and Diaz, 2008; Zou et al., 2017b). In animals, PLCPs are well known lysosomal proteases that perform significant functions in the terminal degradation of proteins within autolysosomes (Kroemer and Jaattela, 2005; Man and Kanneganti, 2016). In recent years, vast majority of plant PLCPs have been characterized during many plant processes. In this review, we summarized what is known of plant PLCPs in growth, seed germination, anther development, senescence, immunity and stress responses (Figure 3 and Table 1).

**Table 1:** Catalog of plant papain-like cysteine proteases (PLCPs) involved in germination, development, senescence, immunity, and stress responses.

PLCP	Species	Function	Reference
EP-B	Barley	Induced expression in the germinating seeds;	Koehler and Ho, 1990;
		Degrade the endosperm storage proteins to provide	Mikkonen et al., 1996.
		nitrogenous nutrients for young seedlings.	
Pap-1	Barley	Involved in grain protein mobilization during germination;	Cambra et al., 2012;
		Silencing decreased germination rate and delayed	Diaz-Mendoza et al., 2016;
		senescence process.	Velasco-Arroyo et al., 2016.
CathB	Arabidopsis	Mutants displayed reduced PCD during abiotic stress and	Ge et al., 2016;
		endoplasmic reticulum stress.	Cai et al., 2018.
Pap-1/6/9	Barley	Inhibition of cathepsins increased the stress-induced	Barany et al., 2018.
		microspore embryogenesis.	
CP14	Tobacco	Silencing delayed PCD of suspensor.	Zhao et al., 2013.
CEP1	Arabidopsis	Mutants delayed tapetal PCD and decreased pollen	Zhang D. et al., 2014
		production.	
SAG12	Arabidopsis	Induced during senescence;	Lohman et al., 1994;
		Mutants decreased yield under low nitrogen (LN) conditions.	James et al., 2018.
*SAG12-H1*RD21	Rubber tree	Highly expressed only in senescent leaves.	Zou et al., 2017a.
1A	Arabidopsis	Involved in dehydration stress; Mutants enhanced susceptibility to *Botrytis cinerea*.	Koizumi et al., 1993; Shindo et al., 2012.
RD19A	Arabidopsis	Involved in dehydration stress; Mutants enhanced susceptibility to *Ralstonia solanacearum*.	Koizumi et al., 1993; Bernoux et al., 2008.
CP	Wheat	Increased expression under abiotic stress and played a role in water deficit; Silencing enhanced tolerance to salt and osmotic stress.	Zang et al., 2010
*CP20.1*	Pepper *Brassica napus*	Ectopic expression leads to premature degradation of tapetum, involvement in tapetum degradation and pollen wall synthesis.	Xiao et al., 2014.Song et al., 2016;
*SPCP2*	Sweet potato	Enhanced resistance to drought and salt stress when overexpressing;Increased sensitivity to drought stress when overexpressing.	Chen et al., 2010;Chen et al., 2013;
Mir1	Maize	Induced expression at wounding site;Enhanced resistance to caterpillar;Acts as ethylene signal conferring resistance to corn leaf aphid;	Chang et al., 2000;Pechan et al., 2002;Louis et al., 2015.
AALP	Arabidopsis Tomato	Increased protein activity in senescent leaves, mutants delay leaf senescence.Up-regulated upon pathogen attack and inhibited by pathogen-derived inhibitors;	Pruzinska et al., 2017.Kruger et al., 2002;Tian et al., 2007;
Rcr3	Tomato	Resistance to *Phytophthora infestans*, *Cladosporium fulvum*, and *Globodera rostochiensis*.	Dixon et al., 2000;Song et al., 2009;Lozano-Torres et al., 2012.
Pip1 C14	*Nicotiana benthamiana*	Silencing plants susceptible to *C. fulvum*, *Pseudomonas syringae*, and *P. infestans*.	Ilyas et al., 2015.Kaschani et al., 2010.
XCP2	Arabidopsis	Silencing plants susceptible to *P. infestans*. Mutants decreased susceptibility to *R. solanacearum*.	Zhang B. et al., 2014.

## PLCPs Function in Seed Germination

Storage, structural, metabolic, and protective proteins are stored in seeds. During germination, these seed proteins were mobilized or degraded to nourish growing seedlings. These processes were

mainly triggered by PLCPs (Grudkowska and Zagdanska, 2004). In germinating maize and wheat, cysteine proteinases amount up to 90% of total proteolytic activity of prolamins (Grudkowska and Zagdanska, 2004). During *Vicia sativa* seed germination, four PLCPs (CPR1, CPR2, proteinase A, and CPR4) were identified with CPR1 and CPR2 were involved in the mobilization of vicilin and 7S storage globulin (Fischer et al., 2000; Schlereth et al., 2000, 2001). 27 PLCPs were identified during barley grain germination (Zhang and Jones, 1995), and two PLCPs (EP-A and EP-B) were purified (Poulle and Jones, 1988). Two isoforms of EP-B (EPB1 and EPB2) were identified in the germinating seeds with its expression was induced by gibberellin (GA) and suppressed by abscisic acid (ABA) (Koehler and Ho, 1990; Mikkonen et al., 1996). The homolog cysteine endopeptidase EP8 of barley EP-A (HvEPA) in triticale was responsible for mobilizing stored proteins during seed germination, and its activity can be inhibited by endogenous cystatin TrcC-4 (Prabucka et al., 2013). During seed germination, these proteases were secreted from the scutellar epithelium and aleurone layer to the endosperm which degrade the endosperm storage proteins to provide nitrogenous nutrients for young seedlings (Mikkonen et al., 1996). In addition, HvPap-1, a GA induced PLCP, was reported to play essential functions in protein mobilization during barley grain germination (Cambra et al., 2012). HvPap-1 was localized to the protein bodies and vesicles in the embryo, and it could degrade barley endosperm proteins (hordeins, albumins, and globulins). The overexpression of *HvPap-1* decreased starch amount in seeds and increased germination rate, while silencing *HvPap-1* displayed an opposite phenotype with increased starch amount in seeds and decreased germination rate (Diaz-Mendoza et al., 2016). These results indicate that PLCPs are important factors in mobilizing storage proteins to promote seed germination, and their expression and/or activity is regulated by GA, ABA, and cystatins. Arabidopsis PLCPs genes display differential expression in different organs, with *PAP2*, *PAP3*, and *RD19A* display high expression in seed, while *PAP4* and *PAP5* display high expression in leaf. These researches highlight divergent functions of different PLCP proteins. In addition, a lot of PLCPs display high expression in various tissues and organs, such as RD21A, RD21B, RD19A, indicating a housekeeping role in plant growth and development (Richau et al., 2012).

In view of the important roles of PLCP proteins in seed germination, the regulation of PLCPs activity should play essential function in seed germination and seedling development. Phytocystatins (PhyCys) are a group of small proteins and can directly inhibit PLCPs activity (Arai et al., 2002). Many PhyCys have been identified, such as TrcC-6 (Siminska et al., 2015) and TrcC-8 protein that exert an inhibitory effect on PLCP through the interaction with PLCPs (Prabucka et al., 2017). The activities of PhyCys and PLCPs need to maintain a relatively balanced level to ensure the normal seed germination (Szewinska et al., 2016).

The nutrients provided by plant seeds are the basis for the growth and development of offspring. PLCPs are one of the key factors to initiate and complete this process (Szewinska et al., 2016). It is well known that seed germination may be destroyed under adversity stresses, resulting in the inability to form seedlings. Under stress conditions, the activity of PLCPs and their regulatory factors may be destroyed, but the specific mechanism of “destruction” and the resulting consequences need further analysis in various plants.

## PLCPs Associated With Programmed Cell Death

Programmed cell death (PCD) is a highly ordered and genetically controlled process that removes unwanted or damaged cells in both eukaryotic and prokaryotic organisms, playing important roles in protecting against environmental stresses and pathogen invasions. DNA fragmentation, reactive oxygen species (ROS) accumulation and organelle “degradation” were general features of PCD process. PCD played essential functions throughout the plant’s life cycle from embryogenesis to plant death (Staal and Dixelius, 2007; Lord and Gunawardena, 2012).

Papain-like cysteine proteases are essential regulators of plant PCD. In Arabidopsis, a lot of PLCPs were reported in the PCD of tracheary element (TE), tapetum, suspensor, and ER-stress-induced cell death, respectively (Zhao et al., 2000; Cai et al., 2018). AtXCP1 and AtXCP2, two xylem-specific PLCPs, were expressed at a high level in xylem during the PCD process. However, no developmental differences were observed in the single *xcp1*, *xcp2* mutants and the double mutant *xcp1*/*xcp2*, suggesting that they act redundantly with other regulators to regulate TE-PCD (Zhao et al., 2000). Cathepsins are the cysteine protease of papain-like C1A and are important regulators involved in numerous plant biological processes, including leaf senescence and PCD. Three Arabidopsis cathepsin B proteins (AtCathB1-3) were identified with tandem mass spectrometry, and its triple mutant displayed a strong reduction in the PCD induced by abiotic stress (e.g., ultraviolet, oxidative stress) and endoplasmic reticulum stress (Ge et al., 2016). Further research showed that the silencing of cathepsin B reduced ROS accumulation and ER-stress-induced PCD (ERSID), while the downregulation of PBA1 increased ERSID, demonstrating that ERSID was positively and negatively regulated by cathepsin B and PBA1, respectively (Cai et al., 2018). High catalytic activity of tobacco PLCP protein NtCP14 was restricted only to the suspensor at the 8- and 32-celled embryo stages, which correlating with the onset of PCD (Zhao et al., 2013). The overexpression of *NtCP14* induces premature cell death in the basal cell lineage and leads to embryonic arrest and seed abortion, whereas the silencing of *NtCP14* leads to profound delay of suspensor PCD (Zhao et al., 2013). These results indicate that PLCPs play key regulatory roles in seed, TE, tapetum, suspension, and ERSID.

Tapetum plays a crucial role in pollen development by secreting numerous nutritive proteins, enzymes, and sporopollenin precursors for pollen maturation (Plackett et al., 2011). Tapetum undergoes PCD during the late stages of pollen development and disruption PCD of tapetal cells will result in male sterility (Ku et al., 2003; Kawanabe et al., 2006; Li et al., 2017). *AtCEP1*, one KDEL-tailed PLCP, is expressed in tapetum and localized in endoplasmic reticulum (Zhang D. et al., 2014). In *cep1* mutant, tapetal PCD was delayed and pollen production was reduced. Further transcriptomic analysis showed that the expression of genes involved in tapetum degradation and pollen development were changed in *cep1* mutant (Zhang D. et al., 2014). These results showed that CEP1 plays an essential function in tapetal cell PCD and pollen development (Zhang D. et al., 2014). Moreover, a papain-like cysteine protease from *Brassica napus*, *BnaC.CP20.1*, is significant to tapetal degeneration and pollen-wall formation (Song et al., 2016). The ectopic expression of *BnaC.CP20.1* prompt tapetum PCD and lead to male sterile (Song et al., 2016). In summary, the tapetum PCD is extremely important for pollen development, and PLCPs play important roles in this process. Although some PLCPs have been studied, the PLCPs which play key roles in the tapetum PCD process have not been identified or studied in depth.

## PLCPs Involved in Leaf Senescence

Leaf senescence is a physiological process that recycling the endogenous nutrients from the senesencing leaves to support the growth of younger leaves and reproductive organs. Protein breakdown is one of the most fundamentally important reactions during leaf senescent and PLCPs play important functions in protein proteolysis during leaf senescence (Bhalerao et al., 2003; Roberts et al., 2012; Diaz-Mendoza et al., 2014). In Arabidopsis, many members of PLCPs, including SAG12, RD21A, AtRD19A, RD19C, ALP/SAG2/AALP/ALEU, CTB1, and CTB3, have been described as participants in leaf senescence (Lohman et al., 1994; Yamada et al., 2001; Gepstein et al., 2003; Guo and Cai, 2004; Guo and Gan, 2014).

SAG12 exhibits a strictly senescence-associated expression pattern in leaves and thus has been widely used as a senescence marker gene (Lohman et al., 1994). The *sag12* mutant did not show any discernible variation in phenotype under normal conditions, whereas, under low nitrogen (LN) conditions, the yield was decreased in *sag12* mutant, suggesting that *SAG12* participate in the N remobilization that sustains seed production. In addition, the *B. napus* homolog of SAG12 and RD21A proteases were also reported in protein degradation and response to N limitation in senescent leaves (Poret et al., 2016).

Based on the senescence-specific characterization of SAG12, an autoregulatory senescence inhibition system (P_SAG12_-IPT) has been explored by fusing the *SAG12* promoter (*PSAG12*) to a cytokinin-biosynthesizing enzyme-isopentenyl transferase (IPT) (Gan and Amasino, 1996). This fusion activates the expression of *IPT* at the onset of senescence and subsequently increases the cytokinin levels which, in turn, delays the decay of plant senescence (Gan and Amasino, 1996). This technology has been successfully applied in practical applications in many plant species and is approaching commercialization (Guo and Gan, 2014). Various *SAG12* orthologs genes with similar functions have beed identified in a variety of plant species including rice, *B. napus*, sweet potato and tobacco (Noh and Amasino, 1999; Chen et al., 2002; Beyene et al., 2006; Gombert et al., 2006; Liu et al., 2010). Similar to Arabidopsis SAG12, rice *P_SAG39_-IPT* transgenic plants displayed a delayed leaf senescence phenotype and greater number of emerged panicles, suggesting the homologs function as Arabidopsis SAG12 (Liu et al., 2010). Similarly, in rubber tree (*Hevea brasiliensis*), there are 17 Arabidopsis *SAG12* orthologs (*HbSAG12H1–17*) with *HbSAG12H1* displayed the same expression pattern as senescence-associated genes, indicating that *HbSAG12H1* can also act as a molecular marker to study the leaf senescence mechanism of *Hevea* (Zou et al., 2017a). Subsequently, Zou et al. (2017b) used *HbSAG12H1* as the indicator to successfully identify six new *PLCP* genes, i.e., *HbRD21B*, *HbRD21E*, *HbRD21F*, *HbCEP1*, *HbXBCP3L*, and *HbRD19B* by deep sequencing of the senescence rubber leaf transcriptome, suggesting that these PLCPs may play important roles in leaf senescence (Zou et al., 2017b). In addition, the individual dark treatment of Arabidopsis leaves (8 weeks old) showed that the leaf senescence program was induced to start, and the activities of many PLCPs were increased, among which RD21A and AALP displayed the highest induction in senescing leaves (Pruzinska et al., 2017). By phenotypic analysis of the *aalp-1* and *rd21A-1*/*aalp-1* mutants, fewer senescent leaves were identified and senescence was delayed than wild type, indicating that AALP may be helpful to the senescence process of plants (Pruzinska et al., 2017).

In summary, leaf senescence is a finely regulated process involving the degradation of many substances. The enzymatic reactions catalyzed by PLCPs encoded by senescence-associated genes (SAGs) are an important pathway for protein degradation. At present, SAG12 is the most intensively studied senescence-associated PLCPs, whose function has been characterized in many species. In addition to SAG12 and other PLCPs that have been studied, there are many more PLCPs participating to leaf senescence remain largely unknown.

## PLCPs Mediate Plant Abiotic Stress Response

Plants are constantly challenged by environmental abiotic stresses (e.g., heat, drought, cold, or salinity). Plants have evolved delicate mechanisms to cope with abiotic stresses by reprogramming the expression of gene subsets and inducing an adaptive response. The recycling of proteins by plant proteolysis is a primary defense line for plant survival. Among protease families, PLCPs are the predominantly up-regulated plant proteases, and exhibit increased expression in response to multiple environmental stresses (Rabbani et al., 2003; Kempema et al., 2007; Roberts et al., 2012; Diaz-Mendoza et al., 2014).

*AtRD21A* and *AtRD19A*, two important protein markers for dehydration stress adaptation, were highly induced by drought and salt stresses (Koizumi et al., 1993). Under PEG, salt and cold stresses, the expression of wheat PLCP gene (*TaCP*) was upregulated (Zang et al., 2010). In addition, transgenic Arabidopsis overexpressing *TaCP* showed stronger drought tolerance under water-stressed conditions than wild-type, indicating that TaCP plays a role in mediating dehydration tolerance (Zang et al., 2010). Sweet potato *SPCP2* gene showed enhanced expression during senescence and was also regulated in response to dark, ABA, JA and ethephon treatment (Chen et al., 2010). The overexpression of *SPCP2* in Arabidopsis enhanced resistance to drought and salt stress (Chen et al., 2010). Whereas, the overexpression of sweet potato *SPCP3* in Arabidopsis conferred sensitivity to drought stress (Chen et al., 2013). The expression of pepper (*Capsicum annuum*) PLCP gene (*CaCP*) was induced during leaf senescence and was also significantly unregulated by abiotic and biotic stress treatments (Xiao et al., 2014). The suppression of *CaCP* in pepper enhanced tolerance to salt and osmotic stress (Xiao et al., 2014). *HvPap-1* was induced in response to dark and nitrogen starvation (Velasco-Arroyo et al., 2016). The overexpression of *HvPap-1* in barley accelerated leaf senescence, while silencing *HvPap-1* with amiRNA delayed senescence process (Velasco-Arroyo et al., 2016). In addition, under stress condition, the expression of three barley cathepsin-like proteins (HvPap-1, HvPap-6, and HvPap-19) were increased and autophagy was activated in barley micropores, and the inhibition of cathepsins by caspase-3 inhibitors reduced apoptosis and increased the stress-induced microspore embryogenesis (Barany et al., 2018). In water deficient barley leaves, cystatin HvCPI-2 and HvCPI-4 delay the natural senescence process and increase tolerance to drought by regulating the expression and activity of HvPap-1, HvPap-12, and HvPap-16 C1A proteases, which may be due to the tight control of protease activity to avoid sudden degradation of proteins (Velasco-Arroyo et al., 2018).

Protein hydrolysis is very important for plants to response adversity stresses. In addition to enhancing plant resistance to stress, many PLCP proteases also accelerate plant leaf senescence or enhance plant sensitivity to abiotic stress. In this case, plants often regulate the gene expression or protein activity of PLCPs through some regulators to promote plant growth and increase crop yield. The currently deep-study regulatory factors of PLCP are phytocystatins, and many researches have revealed that the overexpression of phytocystatins significantly delays plant leaf senescence and increases stress tolerance, and the direct inhibition of protease activity may be the main reason (Kunert et al., 2015; Subburaj et al., 2017; Tan et al., 2017a,b). In conclusion, the differential effects of PLCPs in different species are determined by various factors such as the spatiotemporal pattern of protease expression and action, action substrate, and specific growth period and growth state in which the plant is located. Therefore, in different specific environments, the regulation of PLCPs activity is extremely important to enhance plant stress tolerance.

## PLCPs Play a Key Role in Plant Immunity

In natural environment, plants are also attacked by a diverse array of pathogens and pests, including bacteria, fungi, oomycetes, nematodes, insects, and microbes. Many studies have highlighted the importance of PLCP in defense against plant pathogen. In most cases, a lack of PLCP expression leads to alterations of pathogen resistance because PLCP mutations are more susceptible to pathogen infection (Misas-Villamil et al., 2016).

Maize inbred resistance 1 (Mir1), a secreted maize PLCP that localized in vesicle, showed high accumulation at the wounding site after larval feeding (Chang et al., 2000). Tobacco budworm larvae feeding with Mir1-overexpressing plants caused severe damage to caterpillar for Mir1 degraded the peritrophic matrix of the insect gut (Pechan et al., 2000, 2002). Recent research showed that ethylene (ET) was required for accumulation of *Mir1* and contributed to heighten resistance to corn leaf aphid (CLA) in maize (Louis et al., 2015). In addition, PLCPs are also key regulators of salicylic acid (SA)-dependent defense signaling. Previous studies have shown that SA treatment can strongly induce PLCP protein activity in maize leaves, and the activated apoplastic PLCPs can in turn induce the expression of SA-related immune genes (van der Linde et al., 2012). The latest research reveals that PLCPs need to release *Z. mays* immune signaling peptide 1 (Zip1) through their protein precursors to induce SA accumulation and activate SA defense signaling in leaves (Ziemann et al., 2018). These results indicate that the interaction of SA with PLCPs plays a key role in the expression regulation of downstream defense genes under bioticstress.

Tomato Rcr3 (Required for Cladosporium resistance-3) and Pip1 (Phytophthora inhibited protease-1) were up-regulated upon pathogen challenge and their activities were inhibited by pathogen-derived inhibitors (Kruger et al., 2002; Tian et al., 2007). Deletion of *Rcr3* enhanced plants susceptibility to the pathogen *Phytophthora infestans* (Song et al., 2009), *Cladosporium fulvum* (Dixon et al., 2000), and nematode *Globodera rostochiensis* (Lozano-Torres et al., 2012). Similar with *Rcr3*, the *Pip1* mutant plants were also more susceptible to *C. fulvum*, *Pseudomonas syringae*, and *P. infestans* (Ilyas et al., 2015). Silencing of *C14* in *Nicotiana benthamiana* increased plants susceptibility to *P. infestans* (Kaschani et al., 2010). In addition, Arabidopsis *rd19* and *rd21* mutants are more susceptible to bacterial pathogen *Ralstonia solanacearum* and fungal pathogen *Botrytis cinerea*, respectively (Bernoux et al., 2008; Shindo et al., 2012). Whereas, *xcp2* mutant displayed decreased susceptibility to *R. solanacearum* (Zhang B. et al., 2014).

Taken together, these data demonstrate that PLCPs play a determinative role in regulating pathogen defense. However, as can be seen from the above studies, previous studies have focused on the reduction of plant immunity after mutation of PLCP genes, but there is relatively little understanding of how PLCPs participate in plant immune defenses; furthermore, PLCPs play a key role in the plant defense hormone signaling pathways such as ET- and SA-pathway. We have known that the coordinated interaction among various defensive hormones of plant is crucial for the plant immunity. Therefore, studying the role of PLCPs in various plant hormone pathways and the mechanism of action will be an effective way to understand how PLCPs maintain or enhance plant immunity. On the other hand, synthetically revealing the role of PLCPs in plant immune processes is another important research content for the comprehensive exploration of plant immune mechanisms.

## Concluding Remarks

Over the past few years, the study of plant PLCPs has widened considerably, and deciphering the molecular function of these proteases is advanced. PLCPs have a broad substrate specificity, and their protein location, activation and inactivation are tightly regulated in a number of ways. However, the direct link between PLCPs activation and perception in plant signaling has not been fully explored. Functional redundancy of PLCPs has hampered defining their biological functions. Therefore, multiple experimental approaches including double or even triple mutants are needed to address its biological functions. Clearly, further investigation is required to understand how PLCPs perceive stresses and signals and what is the downstream players in PLCP pathways. Sensitive and novel techniques, such as quantitative proteomics and labeling probes, were used to uncover protease substrates and function (Demir et al., 2018; van der Hoorn and Rivas, 2018), thereby may facilitate to advance our mechanical understanding to the function of plant PLCPs.

## Author Contributions

HL and ZZ conceived and wrote the review. MH, QW, and LC critically reviewed the manuscript. All authors listed approved it for publication.

## Conflict of Interest Statement

The authors declare that the research was conducted in the absence of any commercial or financial relationships that could be construed as a potential conflict of interest.
